# Enhanced Dissolution of Sildenafil Citrate Using Solid Dispersion with Hydrophilic Polymers: Physicochemical Characterization and In Vivo Sexual Behavior Studies in Male Rats

**DOI:** 10.3390/polym13203512

**Published:** 2021-10-13

**Authors:** Mohammed F. Aldawsari, Md. Khalid Anwer, Mohammed Muqtader Ahmed, Farhat Fatima, Gamal A. Soliman, Saurabh Bhatia, Ameeduzzafar Zafar, M. Ali Aboudzadeh

**Affiliations:** 1 Department of Pharmaceutics, College of Pharmacy, Prince Sattam Bin Abdulaziz University, Alkharj 11942, Saudi Arabia; mkanwer2002@yahoo.co.in (M.K.A.); mo.ahmed@psau.edu.sa (M.M.A.); s.soherwardi@psau.edu.sa (F.F.); 2 Department of Pharmacology, College of Pharmacy, Prince Sattam Bin Abdulaziz University, P.O. Box 173, Al-Kharj 11942, Saudi Arabia; g.soliman@psau.edu.sa; 3 Department of Pharmacology, College of Veterinary Medicine, Cairo University, Giza 12211, Egypt; 4 Natural and Medical Sciences Research Center, University of Nizwa, Birkat Al Mauz 616, Nizwa P.O. Box 33, Oman; sbsaurabhbhatia@gmail.com; 5 School of Health Science, University of Petroleum and Energy Studies, Dehradun 248007, Uttarakhand, India; 6 Department of Pharmaceutics, College of Pharmacy, Jouf University, Sakaka 72341, Aljouf Region, Saudi Arabia; azafar@ju.edu.sa; 7 Institut des Sciences Analytiques et de Physico-Chimie pour l’Environnement et les Matériaux, IPREM, UMR5254, CNRS, University Pau & Pays Adour, E2S UPPA, 64000 Pau, France; m.aboudzadeh-barihi@univ-pau.fr

**Keywords:** erectile dysfunction, Kolliphor® P188, Kollidon® 30, Kollidon®-VA64, polymer, sildenafil citrate

## Abstract

Sildenafil citrate (SLC) is a frequently used medication (Viagra®) for the treatment of erectile dysfunction (ED). Due to its poor solubility, SLC suffers from a delayed onset of action and poor bioavailability. Hence, the aim of the proposed work was to prepare and evaluate solid dispersions (SDs) with hydrophilic polymers (Kolliphor® P188, Kollidon® 30, and Kollidon®-VA64), in order to enhance the dissolution and efficacy of SLC. The SLC-SDs were prepared using a solvent evaporation method (at the ratio drug/polymer, 1:1, *w*/*w*) and characterized by Differential Scanning Calorimetry (DSC), Fourier-transform infrared spectroscopy (FTIR), X-ray diffraction (XRD), Scanning electron microscope (SEM), drug content, yield, and in vitro release studies. Based on this evaluation, SDs (SLC-KVA64) were optimized, with a maximum release of drug (99.74%) after 2 h for all the developed formulas. The SDs (SLC-KVA64) were further tested for sexual behavior activity in male rats, and significant enhancements in copulatory efficiency (81.6%) and inter-copulatory efficiency (44.9%) were noted in comparison to the pure SLC drug, when exposed to the optimized SLC-KVA64 formulae. Therefore, SD using Kollidon®-VA64 could be regarded as a potential strategy for improving the solubility, in vitro dissolution, and therapeutic efficacy of SLC.

## 1. Introduction

Erectile dysfunction (ED) is the inability of a male to achieve and maintain erection for a sufficient period of time for satisfactory intercourse with a counterpart female partner [1]. It is also referred to as male impotence. ED is common medical problem that directly affects sexual wellbeing and quality of life. Presently, millions of men around the world have some degree of ED, and more than twice that number are anticipated to be affected by 2025 [2,3]. Men suffer from ED due to the rise in synthetic hormone levels present in our diet/environment and a nutritionally poor and imbalanced diet, resulting in low levels of testosterone formation in the body. 

Sildenafil citrate (SLC) is a potent phophodiasterase-5 inhibitor, marketed under the brand name of Viagra. SLC is an orally administered medication, selectively used to treat ED and pulmonary hypertension (PH). SLC absorbs quickly and acts within 1 h of oral administration, but due to a low aqueous solubility and hepatic first pass metabolism (~ 80% of administered dose), its relative bioavailability is 41% [4,5]. The solubility and bioavailability of SLC can improved by various means, such as, cyclodextrin complex [6,7], orodissovable films [8], dry foam tablets [9], salts and co-crystals [10], Self-nanoemulsifying drug delivery systems (SNEDDS) [11], and spray dried amorphous solid dispersions [12]. The advantages of SD have been mentioned in many investigations, including the improvement of dissolution rate and efficacy of poorly water insoluble drugs [13,14]. 

Solid dispersion (SD) is an efficient approach to improve the solubility and bioavailability of Biopharmaceutical classification system (BCS) II and IV class drugs, which involves dispersion of active ingredients within an inert carrier in a solid state [15,16]. The selection of carrier for the preparation of SDs is very important and directly affects the efficacy and stability of the formulation. Thermodynamically unstable SDs have the tendency to recrystallize into amorphous drug during storage, even with traces of crystalline drug left during preparation and long storage periods [17]. Therefore, complete amorphous SD formation is important, to avoid recrystallization and hence improve the physical properties of the drug. The encapsulation of drugs within hydrophilic polymeric carriers induces better wettability and particle micronization; the main procedure by which SDs improve the solubility and bioavailability of poorly soluble drugs. Hydrophilic polymer carriers play a vital role in increasing the dissolution and bioavalibility of poorly soluble drugs. The function of polymers in formulating SDs is to impart stability and solubility, and modify the dissolution rate. Various polymeric carriers, notably water soluble drug carriers such as polyethylene glycol (PEG) and polyvinylpyrrolidone (PVP), with different molecular weight grades have been used for the preparation of SDs and solid solutions. Cellulose derived natural polymers such as hydroxy propyl methyl cellulose acetate succinate (HPMCAS), ethyl cellulose (EC), and hydroxypropyl methyl cellulose (HPMC) have the desired physicochemical properties, and, hence, they are extensively used in formulating SDs. PEG enables a disordered crystalline state of the drug and forms amorphous interstitial solid solutions by encompassing the drug entity in the interstitial spaces of the polymeric carrier. Glass solution is the term devised for SDs in which hydrophilic polymeric carriers are used to increase the solubility; especially for BCS class II and IV. In order to molecularly dissolve the drug, a large quantity of the hydrophilic polymer is used, which in turn increases the physical instability of the dispersions due to phase separation. The mechanisms involved in the improved solubility and dissolution include the detachment of drug molecules as the hydrophilic carrier dissolves, subsequently forming a supersaturated solution of the drug [18,19,20,21,22]. A study on SDs using a ibuprofen model drug revealed that polymers under the trade names “poloxamer 407” and “poloxamer 188” could increase the solubility and reduce the crystal growth of the drug with their coexistence in the polymeric dispersion, which could have been due to the disruption of the ibuprofen and formation of hydrogen bonds between the drug and polymer [23].

Another polymeric carrier, produced under the trade name Kolliphor® P 188 and commercially available through the BASF corporation, acts as a co-emulsifier in creams, an emulsifier for skin delivery applications, and solid and liquid dispersions [24]. It is a synthetic tri-block copolymer, containing a central hydrophobic chain of polyoxypropylene linked by two hydrophilic chains of polyoxyethylene. The generic name of this nonionic linear copolymer is poloxamer 188 (P188), the letter P indicates the state of the polymer (as a powder). Poloxamer 188 (P188) copolymer has been approved by the FDA as a blood thinner and is used pharmaceutically as a surfactant in toothpastes and mouth washes. The nature of the conforming blocks gives Kolliphor® P 188 amphiphilic and surface active properties, which vary depending on its poly(propylene oxide) and poly(ethylene oxide) contents. Poloxamer 188 containing 80% ethylene oxide acts as a water soluble polymeric carrier, used in solid dispersions to improve the solubility and dissolution rate of poorly water soluble active pharmaceutical ingredients (APIs). Kolliphor® P 188 facilitates solubilization process by micelle formation, in which the drug is enclosed in a hydrophobic core externally covered by a polar hydrophilic head. SDs prepared with ploxamer are reported to enhance the solubility and dissolution rate of the hydrophobic drug, Ebastine [25].

Kollidon® 30 is another water soluble drug carrier (from BASF Germany) that is a polyvinyl-pyrrolidone derivative with a molecular weight of 44,000–54,000 g/mol, transition temperature of 149 °C, and soluble in both aqueous and organic solvents. Commonly called povidon(e), poly(1-vinyl-2-pyrrolidone), povidonum, and polyvidone. Kollidon® 30 is widely used in SDs to improve the solubility and dissolution rate of the drugs by forming a water soluble complex with insoluble drugs. Co-precipitation and co-milling technologies are reported to increase the dissolution rate and bioavailability of water insoluble drugs with the usage of Kollidon® 30. Amorphous solid dispersions (ASDs) prepared by Kollidon® 30 were reported to improve the efficiency of nifedipine [26,27].

A copovidone with an exceptionally high binding capacity, traded as Kollidon® VA 64 and produced by BASF, Germany, has applications as a dry binder for direct compression tableting and as a soluble binder for granulation. These properties make it an attractive and cost-effective alternative to natural binders. Kollidon VA 64 was first used to prepare Lopinavir-Ritonavir combination SDs by Abbott laboratories; thereafter, the solubility of many APIs was increased thanks to copovidone. This copolymer of vinyl-pyrrolidone and vinyl-acetate in a ratio of 6:4 possess a transition temperature (100 °C) and degrades at temperature (230 °C) that allow using APIs of varied polarity and with a wide melting temperature range, and it is extensively employed in hot melt extrusion (HME) and spray drying solid dispersions (SDSDs). Single-phase glassy solutions formed by copovidone, in which API was amorphously lodged and dissolved along with the water soluble carrier, controlling the process polymer, improved the dissolution rate [28,29]. Moreover, copovidone-based SDs have also been found to generate nanoparticles during the process of mass conversion from solid to liquid (dissolution), contributing to the improved solubility and bioavailability of the drug. Recently, Moseson et al. [30] reported that copovidone act as crystal nucleation and growth inhibitor by polymer adsorption on the crystalline drug surface, thus improving the dissolution profile. 

This study focuses on the influence of hydrophilic polymers in the dissolution enhancement of SLC. Here, the prepared SDs transformed the crystalline SLC drug into an amorphous state. The interaction of polymers and SLC in SDs was evaluated and optimized by DSC, FTIR, XRD, SEM, and in vitro release studies. The optimized SD (SLC-K64) showed enhanced dissolution, due to the improve wetting properties of the drug. The SLC-K64 formulae significantly improved the sexual behavior in male rats. The goal of the current study was to develop and optimize a SD that could extend the penile erection in male rats and be a potential approach for the treatment of erectile dysfunction.

## 2. Materials and Methods

### 2.1. Materials

Sildenafil citrate (SLC) was obtained as a gift sample from Jazeera Pharmaceutical Industry (JPI), Riyadh, Saudi Arabia. Kolliphor® P188 (K188), Kollidon® 30 (K30) and Kollidon®-VA64 (KVA64) were received as a gift sample from BASF Co., Ltd. (Ludwigshafen, Germany). All solvents and chemicals used for the study were pure and analytical grade.

### 2.2. Preparation of SLC Solid Dispersion by Solvent Evaporation

**Figure 1:**
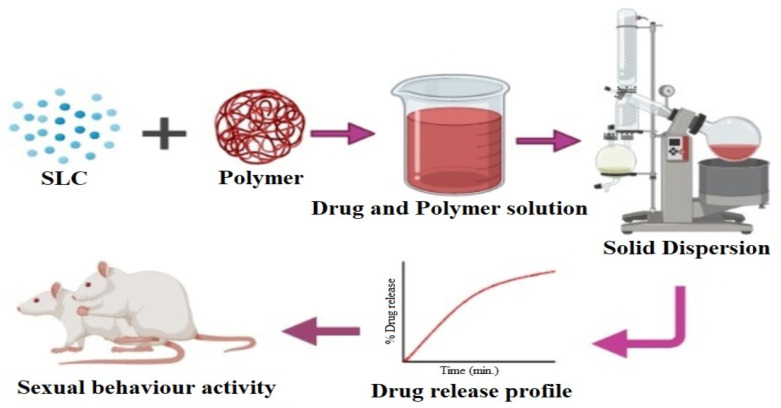
Schematic diagram of preparation of solid dispersion.

The solid dispersions of SLC with each of the polymers (Kolliphor® P188, Kollidon® 30 or Kollidon®-VA64) were prepared (at the ratio drug/polymer, 1:1, *w*/*w*) using a solvent evaporation method [31]. Briefly, an accurately weighed amount of SLC and polymer was dissolved in 60 mL of ethanol and water mixture (1:1, *v*/*v*). The resultant solution was transferred into round bottom flask and evaporated on a rotary evaporator “(Buchi Rotavapor R-215, Essen, Germany)” at 60 °C and 50 rpm for 4 h (Figure 1). The solids retained in the flask were dried under a vacuum overnight to remove residual solvent. The final powder was ground into fine particles and stored for further use. 

### 2.3. Practical Percentage Yield 

The yield of the process was calculated to determine the efficiency of the preparation process. SDs were collected and the percentage yield was estimated using the following equation.

Yield (%) = (Practical weight of the SD)/(Theoretical weight of SLC + Polymer) × 100

### 2.4. Drug Content Estimation 

Drug content was estimated by dissolving 50 mg equivalent weight of SLC in methanol. The solution was then filtered through a syringe filter (0.45 µm), the filtrate was suitably diluted with distilled water and analyzed for the quantity of drug using a UV spectrophotometer at 291 nm against a blank using distilled water (UV-visible spectrophotometer, Jasco 645, Tokyo, Japan). 

### 2.5. Differential Scanning Calorimetry (DSC) Studies

DSC spectra of pure SLC, and their solid dispersions (SLC-K188, SLC-K30, and SLC-KVA64) were recorded using a DSC instrument (SCINCO, DSC N-650, Seoul, Korea) at the temperature range of 50.0–250.0 °C, at a heating rate of 10 °C/min. The instrument was purged with nitrogen gas at a flow rate of 20 mL/min. The DSC apparatus was connected with a sample holder and cooling chamber [32]. Each sample was weighed accurately (approx. 5 mg) and pressed into a hermetically sealed aluminum pan. 

### 2.6. Fourier Transform Infra-Red (FTIR) Spectroscopy 

The FTIR spectra of pure SLC and their solid dispersions (SLC-K188, SLC-K30, and SLC-KVA64) were recorded using an FTIR spectrometer (Jasco FTIR Spectrophotometer, Tokyo, Japan). Each sample was ground with crystalline potassium bromide using a glass mortar and pestle into a very fine particles, and pressed into transparent film. The transparent film was kept on a sample holder and the spectra was recorded using spectra manager software (Jasco, Tokyo, Japan) [33].

### 2.7. Powder X-ray Diffraction (PXRD) Studies

The PXRD pattern of pure SLC and solid dispersions (SLC-K188, SLC-K30, and SLC-KVA64) were recorded with an Ultima IV Diffractometer (Rigaku Inc. Tokyo, Japan at College of Pharmacy, King Saud University, Riyadh, KSA). The set parameters for PXRD were 0–60° (2θ) at a 10°/min scan speed. The anode tube of the instrument used was “Cu with Ka = 0.1540562 nm with mono-chromatized graphite crystal”. The spectra was recorded using a voltage and current of 40 kV and 40 mA, respectively [34].

### 2.8. Scanning Electron Microscopy (SEM)

The morphology of pure SLC, SLC-K188, SLC-K30, and SLC-KVA64 was observed under SEM equipment (JEOL JSM-5900-LV, Tokyo, Japan) operated at 15 KV. The samples were coated with gold using a sputter coater under a vacuum and analyzed using SEM [33].

### 2.9. In Vitro Release Studies

In vitro release studies of SCL from the prepared solid dispersions (SLC-K188, SLC-K30, and SLC-KVA64) compared to pure SLC drug were performed using an USP-2 dissolution apparatus (Fiber optic dissolution system, Model Distek 2500i, Software Rev 1.02, North Brunswick, NJ, USA). Briefly, an accurately weighed sample (equivalent to 50 mg of SLC) was dispersed in a dissolution basket containing 900 mL of phosphate buffer (pH 6.8). The dissolution apparatus was set to run at 100 rpm at 37 ± 0.5 °C. At a predetermined time interval, 5 mL of sample was withdrawn, compensated with fresh media, and analyzed for drug content using UV spectroscopy at 291 nm [35]. Each sample was analyzed in triplicate. 

#### 2.9.1. Mean Dissolution Time (MDT) 

Model independent approaches such as mean dissolution time (MDT) were assessed to study the effect of different polymers within the SDs [36]. The rate of drug dissolved of each SD was expressed by MDT and was calculated using the following equation:

$$MDT=\frac{\sum_{j=1}^{n}t_{j}^{*}\Delta M_{j}}{\sum_{j=1}^{n}\Delta M_{j}}$$

where, (*j*) is the number of the sample (SDs), (*n*) is the number of samples in the dissolution study, (t^*^_j_) is the midpoint time between t and *t* (*j* − 1), and (Δ*Mj*) is the additional amount of drug dissolved between t and *t* (*j* − 1).

#### 2.9.2. Similarity Index (F2)

The similarity index or fit factor of the prepared SDs was assessed, as suggested by Moore and Flanner, comparing the dissolution profiles of the test (SDs) with the reference (SLC). The following equation was used to calculate the *f*_2_ value.

$$f_{2}=50\;X\;log\left\{{\left[1\;+\;(1/n)\;\sum_{t-1}^{n}{\left(Rt-Tt\;\right)}^{2}\right]}^{-0.5}\;\times \;100\right\}$$

where, *f*_2_ means similarity index, *n* stands for dissolution time, and *Rt* and *Tt* denote reference (pure drug) and test (SDs) dissolution values at time *t*. If *f*_2_ values were <50, this suggests a significant difference between the dissolution profiles under comparative study [34,36].

#### 2.9.3. Drug Release Kinetics 

To study the release kinetics, dissolution data were then fit to the drug release kinetic models, and the correlation coefficient (R^2^) was calculated using regression analysis. The zero-order rate describes the concentration independent release kinetics from the SDs, first-order specifies the concentration dependent release rate, the Higuchi̕ model depicts the release of drug based on Fickian diffusion, whereas the Korsmeyer–Peppas model equation demonstrates a relationship between the drug release from the polymeric SDs.

*Qt* = *Q*_0_ + *k*_0_*t* (Zero-order)

*logQ*_*t*_ = *logQ*_0_ − *k*_1_*t* / 2.303 (First-order)

*Qt* = *k*_*H*_*t*^1/2^ (Matrix diffusion)

*Mt*/*M*∞ = *kt*^*n*^ (Korsmeyer–Peppas)

where *Q_t_* (dissolution of drug over time t), *Q_0_* (amount of drug dissolved in diffusion medium at zero time), *k_0_* (zero order constant), *k_1_* (first-order constant), and *k_H_* (Higuchi model constant). *Mt* and *M∞* are the cumulative drug release at time *t* and infinite time, respectively; *k* is the rate constant of drug-polymer particle’s feature, *t* is the release time. Diffusional exponent (*n*) indicates the drug release mechanism. When *n* = 0.45 (Case I or Fickian diffusion), 0.45 < *n* < 0.89 (anomalous behavior or non-Fickian transport), *n* = 0.89 (Case II transport) and *n* > 0.89 (Super Case II), based on the exponent value release mechanisms reported.

### 2.10. In Vivo Sexual Behavior Studies

#### 2.10.1. Animals

Male (250–300 g) and female albino rats (150–200 g) were used for the sexual behavior study [37]. All animals were bred in the lab care unit at the College of Pharmacy, Prince Sattam Bin Abdulaziz University, Alkharj, Saudi Arabia. Rats were kept in separate cages with access to food and water ad libitum. The study protocol was reviewed and approved by the “Animal Ethics Committee (Approval number: BERC 005-05-19), College of Pharmacy, Prince Satam Bin Abdulaziz University, Alkharj, Saudi Arabia”.

#### 2.10.2. Preparation of Male and Female Rats

The male rats were trained sexually with receptive females three times a day for four days before commencement of the experiment. The male rats that did not show any sexual activity during training were excluded from experiment. Eighteen sexually active male rats were selected for the sexual activity of sildenafil citrate and its optimized solid dispersion. The female rats were made receptive by administering estradiol benzoate (10 mg/kg body weight) and progesterone (1.5 mg/kg body weight) subcutaneously, 48 h and 4 h prior to pairing with male rats, respectively. The sexual activity of the female rats was confirmed prior to the test by exposing them to male rats. The most receptive female rats were marked and selected for the study.

#### 2.10.3. Experimental Procedure

The aphrodisiac experiments were performed as per a previously reported method [35]. Eighteen healthy and sexually active male rats (250–300 g) were selected for the study. They were divided into three groups of 6 animals, each group was isolated alone in separate cages during the study. Group I (Control) received 1% *w*/*v* sodium carboxymethyl cellulose (Na-CMC) as a vehicle at rate of 5 mL/kg. Group II (reference) received SLC at a dose of 5 mg/kg, and, finally, group III (optimized formulation) received formulation (equivalent to 5 mg/kg SLC pure). The control vehicle, SLC and formulation were administered as a single dose by orogastric cannula one hour before the start of the study.

#### 2.10.4. Monitoring of Sexual Behavior

The most sexually active female rats were selected for the study. The experiment was performed at 19:00 h in a noiseless room under dim red light in transparent cages. The single female rat was introduced into the cage of single male rat for 15 min, considered as an adaptive period, and after this period, the females were separated from the male cages, then control (Na-CMC), SLC suspension, and formulation were administered orally. The treated female rat was again paired with same male rat in the cage, and the sexual behavior of the male rat was immediately started and continued for the first two matings. The following sexual behavior parameters were monitored and noted as described in a previous study [35,38].

The following definitions were considered for this test: mounting latency (ML), the time from the pairing of the female and male in one cage and first mount; intromission latency (IL), the time from the pairing of a female and male in one cage and first intromission (vaginal penetration) by the male; mount frequency (MF), the number of mounts before ejaculation, that is lifting of the male’s fore body over the hind body of the female and clasping her flanks with his forepaw; intromission frequency (IF), the number of vaginal penetrations before ejaculation; ejaculation latency (EL), the time from the first vaginal penetration of a series to the ejaculation; post-ejaculatory interval (PEI), the time from ejaculation to the first vaginal penetration of the next copulatory series. In the second mating only the EL was recorded. Percentage copulatory efficiency (%CE) was calculated using the following equation:

$$\begin{array}{c} \;\%\mathrm{CE}=\;\mathrm{IF}/\mathrm{MF}\;\times 100\\ \%\mathrm{ICE}=\;\mathrm{IF}/\mathrm{IF}+\mathrm{MF}\;\times 100 \end{array}$$

### 2.11. Statistical Evaluation

The significance of difference between the means was determined by one-way analysis of variance (ANOVA) with a post-hoc test. A *p*-value < 0.05 was considered significant.

## 3. Results and Discussion

### 3.1. Practical Percentage Yield 

The percentage yield of prepared SDs was determined, to ascertain the loss during the solvent evaporation process. SDs prepared by solvent evaporation showed percentage yield values ranging between 93 and 95.8%. The high percentage yield indicates the minimum loss, homogeneity, and accuracy of the process, and hence the suitability for scale-up.

### 3.2. Drug Content Estimation of Solid Dispersions

Drug content estimation determined the uniformity of the drug in the polymeric dispersion, the value of drug estimation was found to be in the range of 97–98.99%. 

### 3.3. DSC Studies

**Figure 2:**
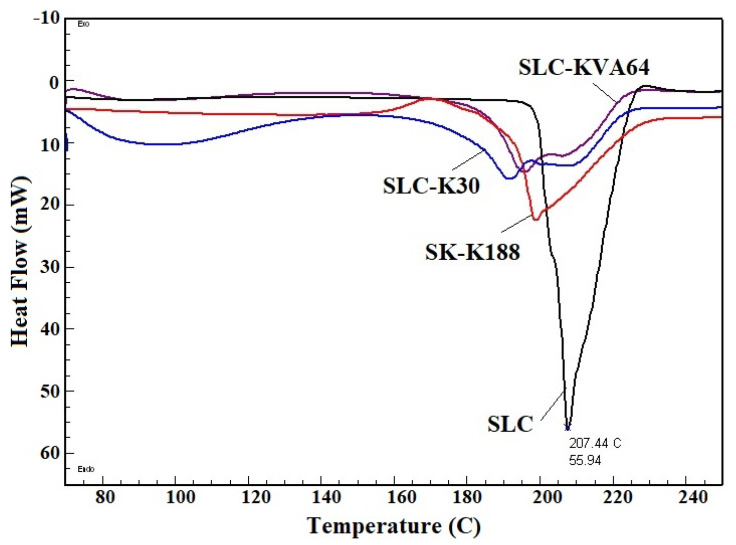
Comparative DSC thermogram of pure SLC, SLC-K188, SLC-K30, and SLC-KVA64.

DSC is a thermal analytical technique used in formulation development to understand the physicochemical properties of pure drugs and their formulations. The presence of crystallinity of pure SLC was detected by DSC spectra, because of a sharp endothermic peak at 207 °C (Figure 2), which was close to the previous reported data [39]. The DSC peaks indicate that SLC does not have any exothermic/degradation peaks, confirming its stability up to a temperature of 250 °C. The solid dispersions SLC-K188 and SLC-K30 showed a broad endothermic peak at 198 °C and 192 °C with reduced intensity, respectively, indicating their partial crystallinity. However, SLC-KVA64 showed the complete disappearance of endothermic peaks corresponding to SLC drug peaks, indicating the transformation of crystalline phase to amorphous phase, due to dissolution into the KVA64 polymer matrix. The SLC drug did not crystallize in the SLC-KVA64 system, due to complete dispersion in the polymer matrix.

### 3.4. FTIR Studies

**Figure 3:**
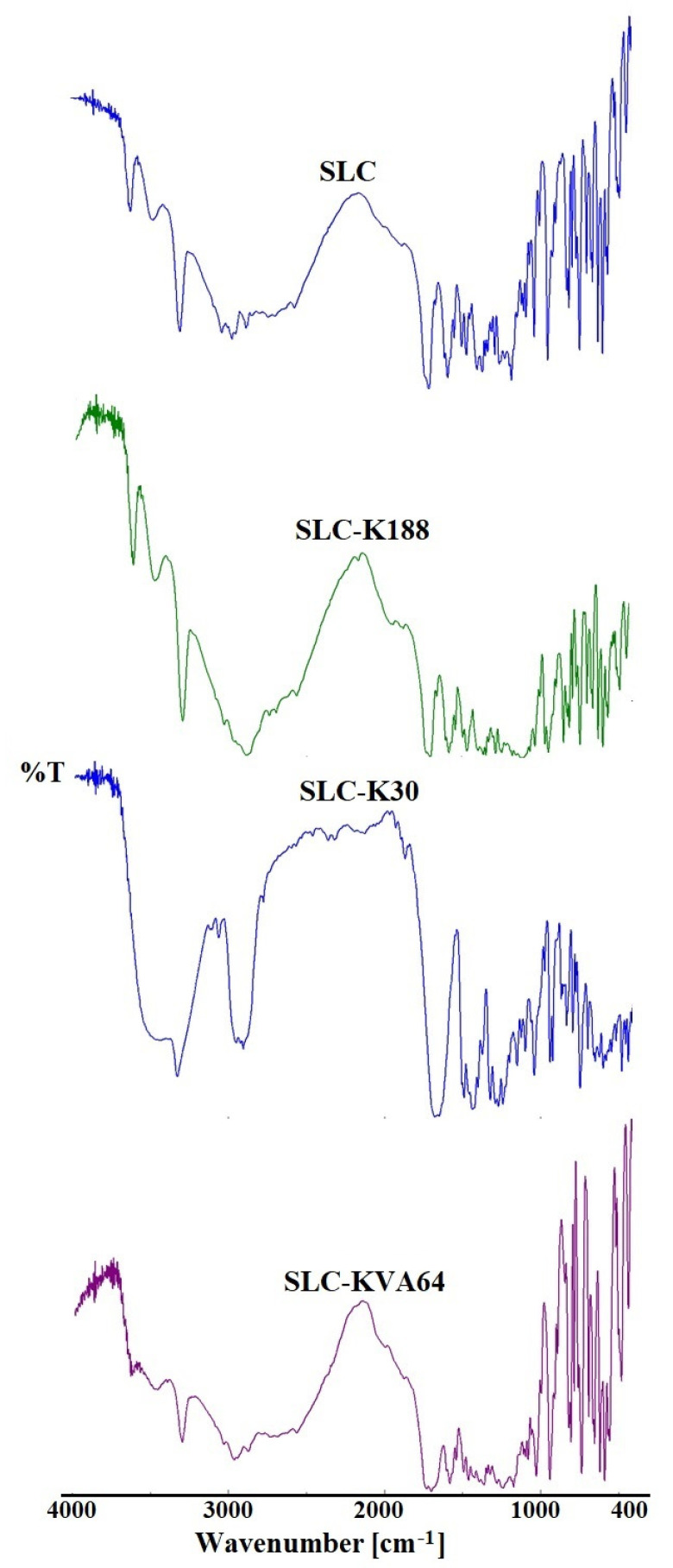
Comparative FTIR spectra of pure SLC, SLC-K188, SLC-K30, and SLC-KVA64.

FTIR spectra of pure SLC and solid dispersions SLC-K188, SLC-K30, and SLC-KVA64 are presented in Figure 3. The pure SLC showed characteristic peaks in frequency at 1170 cm^−1^ and 1265 cm^−1^ for asymmetric and symmetric SO_2_ bands. A strong peak at 1495 cm^−1^ could be assigned to the –COOH group present in citric acid. The two strong peaks could be attributed to the –N–H bend and –N–H stretching at frequency 1582 cm^−1^ and 3301 cm^−1^; these assigned peaks confirmed the purity of the drug [35,40]. Compared to the pure SLC, the characteristic peak of SLC was absent or weakened in the prepared solid dispersions (SLC-K188, SLC-K30, and SLC-KVA64), confirming the successful dispersion of polymers with drug [41].

### 3.5. PXRD Studies

**Figure 4:**
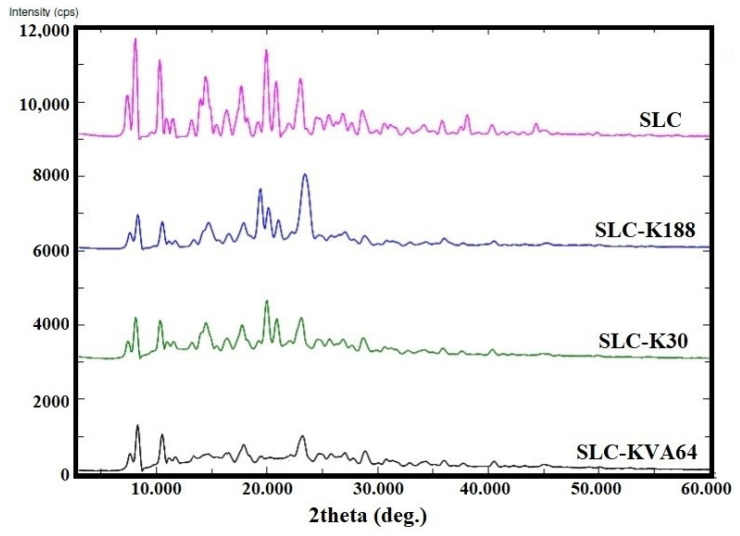
Comparative PXRD spectra of pure SLC, SLC-K188, SLC-K30, and SLC-KVA64.

PXRD spectral analysis is a useful tool for identifying the crystalline/amorphous nature of a solid state powder. The PXRD patterns of pure SLC, SLC-K188, SLC-K30, and SLC-KVA64 are shown in Figure 4. The PXRD pattern of pure SLC revealed several diffraction peaks between 0 and 60° (2θ), which confirmed the crystalline nature of the drug [37,41]. The solid dispersions SLC-K188 and SLC-K30 showed a few diffraction peaks corresponding to SLC with reduced intensity, indicating partial crystallinity. However, the PXRD pattern of the SD, SLC-KV64, showed a typical profile of an amorphous compound, suggesting that the polymer KVA64 inhibited the drug crystallization by reordering of the crystal lattice; this findings strongly supports the DSC analysis [42]. The amorphous powder influenced the faster dissolution of the drug, due to increased internal energy and molecular motion, which improved the thermodynamic property as compared to the pure SLC crystalline drug. The reduction of crystallinity of the drug could have been due to dispersion of the polymers (K188, K30, and KVA64) with the SLC drug [43].

### 3.6. SEM

**Figure 5:**
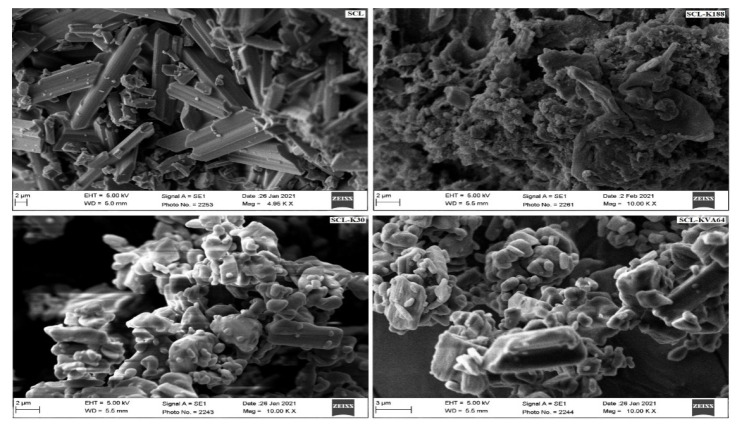
SEM images of pure SLC, SLC-K188, SLC-K30, and SLC-KVA64.

Crystalline and amorphous solid dispersion can be differentiated visually using SEM images. According to the SEM images, the high crystallinity of SCL evidenced a large needle shaped powder. The SEM images of SDs (SLC-K188, SLC-K30, and SLC-KVA64) did not show any crystalline structure and the SDs appeared as aggregates of irregular shape. As can be seen, these polymers strongly disrupted the morphology of the SDs, due to transformation from a crystalline to amorphous state (Figure 5). The crystallinity of SLC was already confirmed by the XRD and DSC studies.

### 3.7. In Vitro Release Studies

**Figure 6:**
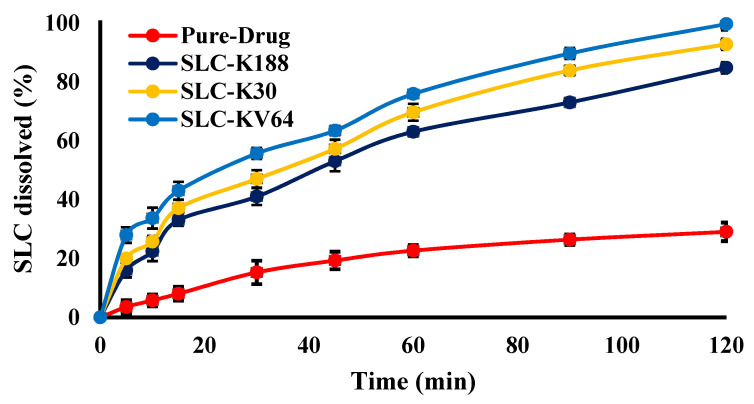
In vitro release profile of pure SLC, SLC-K188, SLC-K30, and SLC-KVA64.

Researchers have explored solid dispersion/inclusion complexation to improve the solubility, permeability, oral bioavailability, and therapeutic effectiveness of SLC through its encapsulation with polymers, with the aim of achieving an enhanced drug release [8]. In vitro release profiles of SLC from the prepared solid dispersions (SLC-K188, SLC-K30, and SLC-KVA64) are presented in Figure 6. Enhanced dissolution of SLC was noted in all solid dispersions. The pure SLC drug showed a much lower and incomplete release (29.08%) during the time-scale of the study (2 h). However, an almost complete release of SLC was observed from all the prepared solid dispersions. The maximum drug release was recorded by the SLC-KVA64 system (99.74%) after 2 h. The improvement in dissolution rate of SLC in a solid dispersion can be attributed to the dispersion of hydrophilic polymers, due to the wettability of the drug, which resulted in an enhancement in solubility [44]. These results suggest that the use of hydrophilic polymers as the carrier transformed the SLC crystals into an amorphous state, which improved the solubility of the drug. However, the highest release of SLC from SLC-KVA64 could possibly have been due to the maximum amorphization of SLC, wetting properties of SLC-KVA64 polymer with SLC.

The estimated values of similarity factor (f2) and MDT for all solid dispersions are summarized in Table 1. It was found that the f2 values for all formulae (SLC-K188, SLC-K30, and SLC-KVA64) were less than 50, suggesting all formulae are statistically not different (*p* > 0.05). The mean dissolution time is an indication of the dissolution process. The MDT for SLC-K188, SLC-K30, and SLC-KVA64 were estimated as 39.61, 37.65, and 35.52 min. The lowest MDT was measured for SLC-KVA64 among all the formulae, indicating a faster release of drug compared to the other formulae. However, the release kinetic models of fitted f and coefficient of correlation (R^2^) values were obtained as first order (R^2^ = 0.999), matrix diffusion (R^2^ = 0.996), and matrix diffusion (R^2^ = 0.998) for the formulae SLC-K188, SLC-K30, and SLC-KVA64, respectively.

### 3.8. In Vivo Sexual Behavior Studies

**Figure 7:**
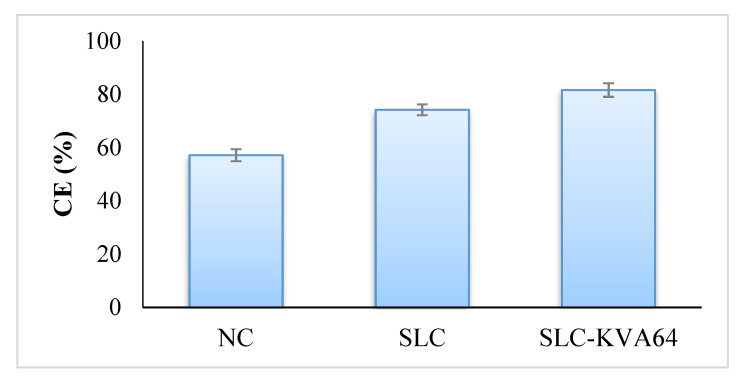
Effect of pure SLC and SLC-KV64 on CE.

**Figure 8:**
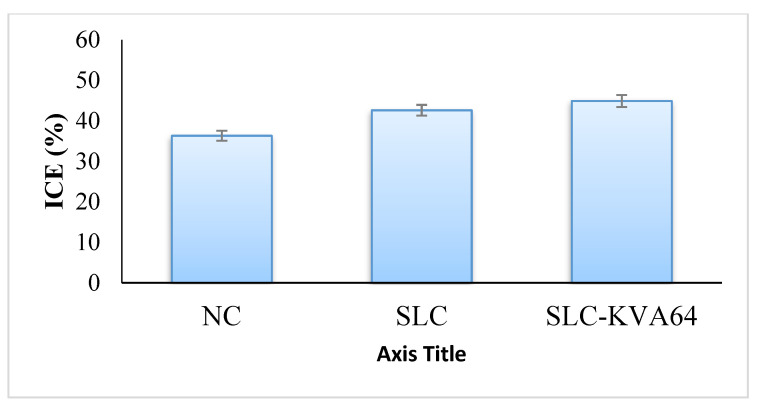
Effect of pure SLC and SLC-KV64 on ICE.

In this study, the SLC-KV64 formulation was tested and compared with pure SLC for its aphrodisiac effect on male rats. The male rats showed an improved sexual activity towards their female rat partner, shown by their eager and quick movement and visible signs of pre-copulatory action, such as anogenital exploration, body sniffing, and moving around, which finally resulted in mounting [35]. The data presented in Table 2 and Table 3 show that SLC (5 mg/kg) significantly reduced the ML (67.27 ± 2.18 s) and IL (108.15 ± 3.52 s) and caused a significant increment in the MF (8.57 ± 0.37) and IF (5.36 ± 0.36) compared to the control. MF and IF are important indicators to measure the vigor, libido, and potency and that reflect the sexual motivation and efficiency of erection, respectively. The increase in MF and IF following administration of SLC-KV64 formulation was observed, suggesting the improved sexual behavior of rats [45]. As ML and IL values decreased following administration of SLC-KV64, this suggests stimulation of sexual motivation and arousal [46]. One formulation (SLC-KV64) also prolonged ejaculatory latency in the first and second series (EL-1 and EL-2) and caused a significant reduction in the post ejaculatory interval (PEI) compared to the control group. Due to EL and PEI events, the refractory period between the first and second series of mating, revealed that SLC in the formulation improved the sexual activity. The CE (%) and ICE (%) are presented in Figure 7 and Figure 8. Improvements in CE (81.6%) and ICE (44.9%) were observed when rats were exposed to optimized SLC-KVA64 formulae in comparison to the pure SLC drug [37]. All observed sexual activity parameters of the optimized SLC-KVA64 were remarkably improved compared to the control and pure SLC drug; these parameters are statistically significant.

## 4. Conclusions

In this study, SDs of SLC were prepared by solvent evaporation method using three hydrophilic polymers as drug carriers, namely Kolliphor® P188, Kollidon® 30, and Kollidon®-VA 64. The prepared SDs succeeded in improving the dissolution rate and sexual behavior in male rats. A marked influence of the polymers on SCL dissolution was noted. All SDs significantly improved the SCL dissolution compared to the pure drug. The optimized SD (SLC-KV64) system showed the maximum enhancement in dissolution rate compared to the pure SLC drug. The DSC and PXRD studies revealed the transformation of the crystalline state of SLC to an amorphous state, which required the lowest energy for drug solubilization. A significant improvement in sexual activity was observed in optimized SD (SLC-KV64) administered male rats, compared to pure SLC drug. Finally, we concluded from this research that the optimized SDs (SLC-KVA64) exhibited superior activity and are a promising strategy for improving solubility, dissolution rate, and aphrodisiac effects on male rats. Hence, the results suggest that the hydrophilic polymer Kollidon®-VA 64 could be an excellent carrier for enhancing the dissolution and therapeutic performance of SLC drug.

## Figures and Tables

**Table 1:** Similarity factors and release kinetics of prepared SDs.

Solid Dispersions	MDT (min)	f2	Correlation Coefficient (R^2^)				
			Zero Order	First Order	Matrix Diffusions	Korsmeyer-Peppas	N
SLC-K188	39.61	25.22	0.910	0.999	0.996	0.991	0.524
SLC-K30	37.65	21.68	0.907	0.989	0.996	0.992	0.495
SLC-KVA64	35.52	18.59	0.876	0.840	0.998	0.995	0.411

**Table 2:** Effect of SLC-KV64 on the mount latency (ML), mount frequency (MF), intromission latency (IL), intromission frequency (IF), and copulatory efficiency (CE) of male rats.

Groups	ML (s)	MF	IL (s)	IF
NC	122.71 ± 5.27	4.24 ± 0.48	245.15 ± 7.27	2.42 ± 0.15
SLC-STD	67.27 ± 2.18 *	8.57 ± 0.37 *	108.15 ± 3.52 *	5.36 ± 0.36 *
SKV64	59.16 ± 2.11 *ǂ	10.36 ± 0.42 *ǂ	97.78 ± 3.16 *ǂ	8.45 ± 0.50 *ǂ

Values are expressed as mean ± S.E.M., *n* = 6 rats/group. * indicates significance compared to NC group at *p* < 0.05. ǂ indicates significance compared to SLC-STD group at *p* < 0.05.

**Table 3:** Effect of SLC-KV64 on the ejaculation latency in the 1st series (EL-1), post ejaculatory interval (PEI) and ejaculation latency in the 2nd series (EL-2) of male rats.

Groups	EL-1 (s)	PEI (s)	EL-2 (s)
NC	376.62 ± 8.72	496.64 ± 14.73	395.20 ± 7.12
SDL-STD	423.16 ± 11.46 *	397.84 ± 8.46 *	421.22 ± 8.25 *
SKV64	458.38 ± 10.27 *ǂ	370.50 ± 8.50 *ǂ	449.20 ± 9.28 *ǂ

Values are expressed as mean ± S.E.M., *n* = 6 rats/group. * indicates significance compared to NC group at *p* < 0.05. ǂ indicates significance compared to SLC-STD group at *p* < 0.05.

## Data Availability

The data presented in this study are available on request from the corresponding author.
